# SLC4A11 mediates ammonia import and promotes cancer stemness in hepatocellular carcinoma

**DOI:** 10.1172/jci.insight.184826

**Published:** 2024-11-08

**Authors:** Ameer L. Elaimy, Marwa O. El-Derany, Jadyn James, Zhuwen Wang, Ashley N. Pearson, Erin A. Holcomb, Amanda K. Huber, Miguel Gijón, Hannah N. Bell, Viraj R. Sanghvi, Timothy L. Frankel, Grace L. Su, Elliot B. Tapper, Andrew W. Tai, Nithya Ramnath, Christopher P. Centonze, Irina Dobrosotskaya, Julie A. Moeller, Alex K. Bryant, David A. Elliott, Enid Choi, Joseph R. Evans, Kyle C. Cuneo, Thomas J. Fitzgerald, Daniel R. Wahl, Meredith A. Morgan, Daniel T. Chang, Max S. Wicha, Theodore S. Lawrence, Yatrik M. Shah, Michael D. Green

**Affiliations:** 1 Department of Radiation Oncology and; 2 Department of Molecular and Integrative Physiology, University of Michigan, Ann Arbor, Michigan, USA.; 3 Department of Biochemistry, Faculty of Pharmacy, Ain Shams University, Cairo, Egypt.; 4 Graduate Program in Immunology, University of Michigan, Ann Arbor, Michigan, USA.; 5 Cayman Chemical Company, Ann Arbor, Michigan, USA.; 6 Department of Medicine, Herbert Irving Comprehensive Cancer Center, Columbia University Medical Center, New York City, New York, USA.; 7 Department of Surgery and; 8 Division of Gastroenterology, Department of Internal Medicine, University of Michigan, Ann Arbor, Michigan, USA.; 9 Gastroenterology Section, Department of Internal Medicine, Veterans Affairs Ann Arbor Healthcare System, Ann Arbor, Michigan, USA.; 10 Department of Microbiology and Immunology,; 11 Division of Hematology and Oncology, Department of Internal Medicine, and; 12 Department of Radiology, University of Michigan, Ann Arbor, Michigan, USA.; 13 Division of Oncology and; 14 Department of Radiation Oncology, Veterans Affairs Ann Arbor Healthcare System, Ann Arbor, Michigan, USA.; 15 Department of Radiation Oncology, UMass Chan Medical School, Worcester, Massachusetts, USA.

**Keywords:** Metabolism, Oncology, Amino acid metabolism, Liver cancer, Oncogenes

## Abstract

End-stage liver disease is marked by portal hypertension, systemic elevations in ammonia, and development of hepatocellular carcinoma (HCC). While these clinical consequences of cirrhosis are well described, it remains poorly understood whether hepatic insufficiency and the accompanying elevations in ammonia contribute to HCC carcinogenesis. Using preclinical models, we discovered that ammonia entered the cell through the transporter SLC4A11 and served as a nitrogen source for amino acid and nucleotide biosynthesis. Elevated ammonia promoted cancer stem cell properties in vitro and tumor initiation in vivo. Enhancing ammonia clearance reduced HCC stemness and tumor growth. In patients, elevations in serum ammonia were associated with an increased incidence of HCC. Taken together, this study forms the foundation for clinical investigations using ammonia-lowering agents as potential therapies to mitigate HCC incidence and aggressiveness.

## Abstract 2

This manuscript demonstrates that ammonia contributes to hepatocellular carcinoma initiation by serving as a nitrogen resource for tumor initiating cells.

## Graphical abstract

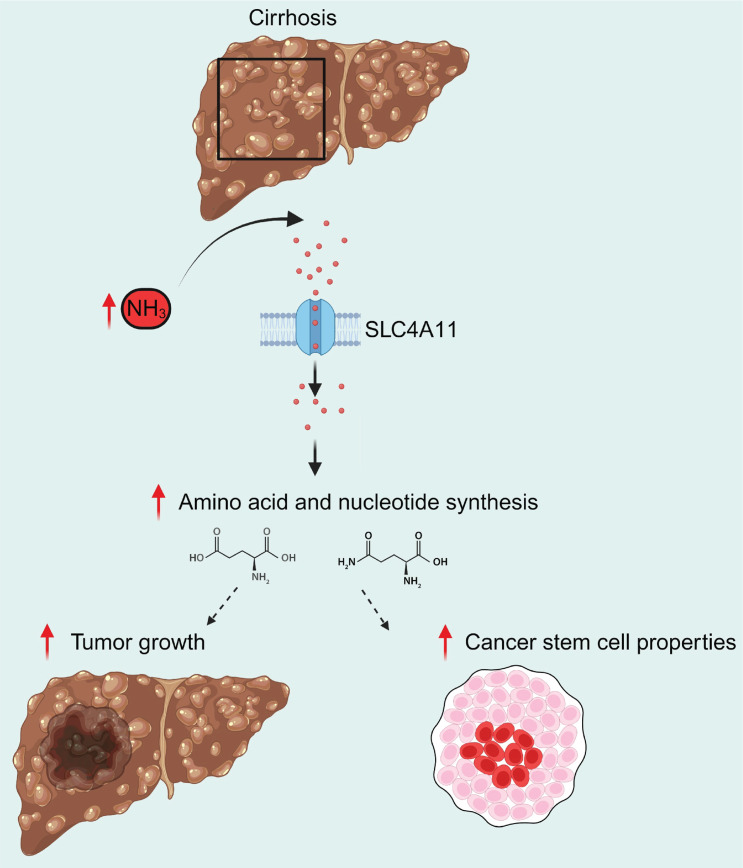

## Introduction

The vast majority of hepatocellular carcinoma (HCC) cases arise in the setting of chronic liver disease and cirrhosis (1). Given the high risk of developing HCC in patients with cirrhosis, screening programs are recommended to detect tumors at an early stage and guide clinical decision making (2). Most patients with liver-confined HCC are not candidates for potentially curative resection or transplant due to poor functional status, inadequate hepatic reserve, or tumor location (3–6). These patients are subsequently evaluated for various liver-directed therapies (7). Although local treatments are generally effective in providing tumor control, the development of new intrahepatic lesions is common (8). Cell damage from oxidative stress caused by chronic inflammation is thought to contribute to the high propensity to initiate tumors in patients with cirrhosis (9–11). However, a greater understanding of the effect of the liver microenvironment on HCC initiation is needed with the goal of exploiting the pathophysiological consequences that result from liver dysfunction for therapeutic benefit.

Ammonia is a nitrogenous waste product of amino acid metabolism that undergoes detoxification by the liver via the urea cycle (12). In patients with cirrhosis, inadequate ammonia clearance contributes to neuropsychiatric sequelae, including hepatic encephalopathy (13). While well recognized as a toxic metabolic byproduct, some malignancies can metabolically recycle ammonia for incorporation into anabolic pathways that promote tumor growth and contribute to therapy resistance (14, 15). These observations are of particular relevance to HCC as it arises in a background of cirrhosis (16). However, whether ammonia contributes to HCC initiation and progression remains unknown.

Cancer stem cells (CSCs) are a subpopulation of cells that are responsible for tumor initiation, therapy resistance, and disease recurrence (17, 18). CSCs reside in a niche of immune and stromal cells, extracellular matrix contacts, and secretory molecules that, together, support self-renewal (19). HCC is enriched in CSCs that are adapted to survive in the unique microenvironment of a cirrhotic liver (20–23). In this study, we explored the interplay between metabolic alterations in cirrhosis and hepatocellular carcinogenesis using preclinical models and patient data. We discovered that ammonia contributed to tumor initiation by serving as a precursor for amino acid and nucleotide biosynthesis in CSCs and that elevated ammonia correlated with cancer incidence and poor prognosis in patients with HCC. Our work suggests that targeting this crosstalk may reduce the incidence and aggressiveness of HCC in patients with chronic liver disease.

## Results

### Elevated ammonia is associated with an increased incidence of and poor prognosis in HCC.

**Figure 1:**
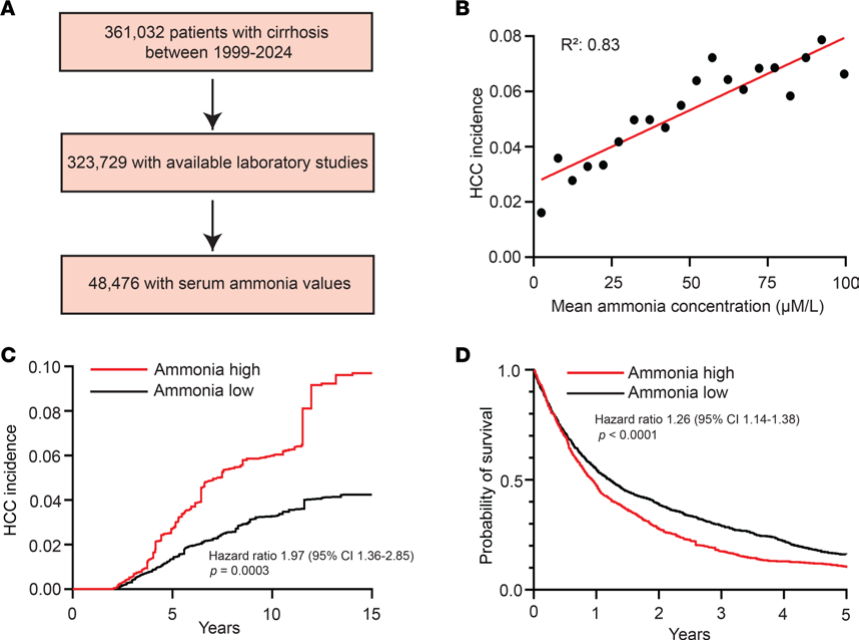
Elevated ammonia is associated with an increased incidence of and poor prognosis in HCC. (**A**) CONSORT diagram of patient population. (**B**) Scatter plot depicting correlation between mean ammonia concentration and HCC incidence fit using linear regression of 95% of the 48,476-patient cohort with mean ammonia concentrations ≤ 100 μM/L. (**C**) Two-year landmark analysis of HCC incidence in patients with high and low ammonia using propensity score matching. (**D**) OS of patients with high and low ammonia using propensity score matching. Hazard ratio log-rank test, *P* values, and 95% confidence intervals indicated.

We and others have shown that ammonia can support tumor growth in breast and colorectal cancer (14, 15). It remains unclear whether hepatic insufficiency and longitudinal elevations in ammonia contribute to hepatocellular carcinogenesis. To examine this question, we identified a cohort of 363,032 patients with cirrhosis diagnosed between 1999 and 2024. Approximately 48,476 patients within this cohort had quantification of serum ammonia levels at the time of cirrhosis diagnosis (Figure 1A) (Cohort 1). We first asked whether elevations in baseline ammonia were associated with an increased risk of developing HCC. Interestingly, we observed a significant positive association between baseline ammonia levels and HCC incidence (Figure 1B). To evaluate this association more rigorously, we next stratified patients by baseline ammonia level into those patients with high and low mean ammonia values (ammonia high ≥ 48.5 μM/L, median = 69 μM/L; ammonia low < 48.5 μM/L, median = 31 μM/L) (24). Patients with cirrhosis with high and low ammonia were similar in terms of age, ethnicity, and etiology of their underlying liver disease (Table 1). We observed that patients with high baseline ammonia had a significantly higher cumulative incidence of HCC (HR 2.02 [95% CI, 1.86–2.20], *P* < 0.0001) as compared with patients with low ammonia levels (Supplemental Figure 1A; supplemental material available online with this article; https://doi.org/10.1172/jci.insight.184826DS1).

To understand whether elevations in ammonia correlated with increased cumulative incidence of HCC in all subsets of patients, we performed additional analyses. Albumin-bilirubin (ALBI) grade (25), model for end-stage liver disease (MELD) (26), and Child-Turcotte-Pugh (CTP) class (27) are widely utilized assessments of the degree of liver dysfunction in patients with cirrhosis. We observed that elevations in baseline ammonia correlated with increased HCC incidence regardless of the ALBI grade, MELD score, and electronic CTP (eCTP) class (Supplemental Figure 1B). To more rigorously evaluate this association, we next conducted a propensity score weighted landmarked analysis. Multivariable analysis showed that the cumulative incidence of HCC was similar in patients with cirrhosis who had ammonia quantified as compared with those patients who did not have ammonia quantified (Supplemental Figure 1C). In contrast, multivariable analysis showed that the cumulative incidence of HCC remained significantly higher in patients with elevated ammonia as compared with those patients without elevations after correction for clinicopathologic features (HR 1.97 [95% CI, 1.36–2.85], *P* = 0.0003) (Figure 1C). Together, these data suggest that elevations in serum ammonia in patients with cirrhosis may be associated with a higher risk of developing HCC.

We next hypothesized that elevations in ammonia may be associated with adverse outcomes in patients with HCC. To evaluate this, we identified a cohort of 3,550 patients with a diagnosis of HCC from the cancer registry and similarly stratified patients with high and low ammonia (Cohort 2). Patients with HCC with high and low ammonia were similar in terms of age, ethnicity, and etiology of their underlying liver disease, and patients with high ammonia had worse overall liver function as measured by ALBI grade, MELD score, and eCTP class (Table 2). On univariate analysis, we observed that patients with elevated ammonia had a worse overall survival (OS) (HR 1.29 [95% CI, 1.20–1.38], *P* < 0.0001) (Supplemental Figure 2A). On subset analysis, patients with elevated ammonia had significantly inferior OS in the subset of patients with relatively well-preserved liver function (ALBI grade 1, MELD ≤ 9, and eCTP class A) (Supplemental Figure 2B). There was a trend toward inferior OS in those patients with moderate liver function and elevated ammonia (ALBI grade 2, MELD score 10–19). Multivariable modeling using propensity score weighting confirmed that patients with elevated ammonia had a worse OS (HR 1.26 [95% CI, 1.14–1.38], *P* < 0.0001) (Figure 1D). These data suggest that elevated ammonia is also associated with poor prognosis in patients with HCC.

### Ammonia contributes to CSC function and HCC initiation.

**Figure 2:**
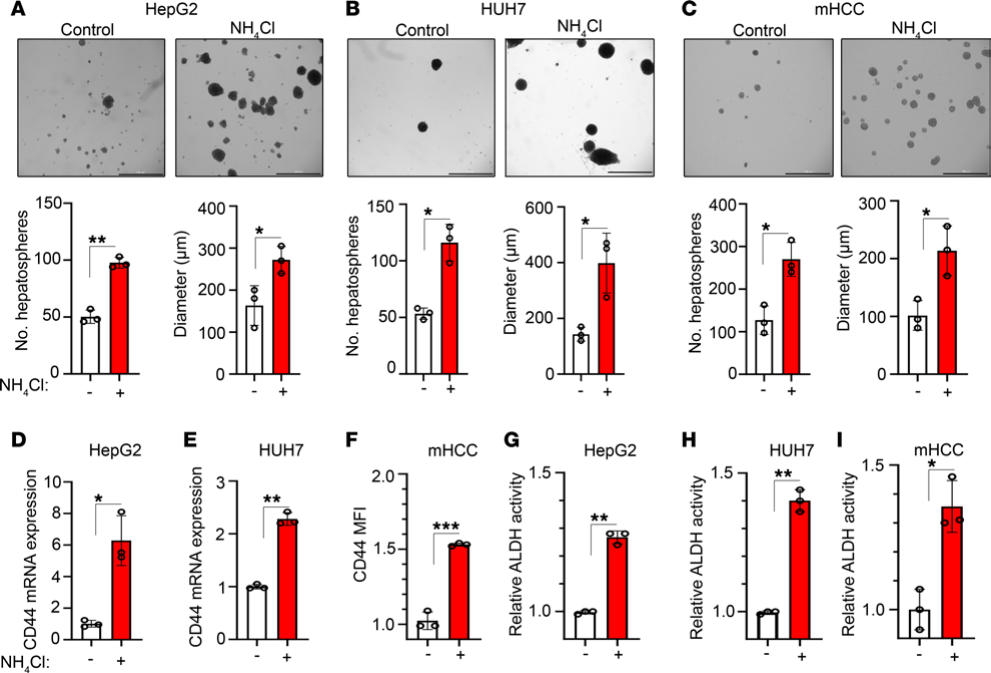
Ammonia promotes the acquisition of cancer stem cell properties in vitro. (**A**–**C**) Representative bright-field micrographs, number of hepatospheres, and diameter of hepatospheres formed with and without ammonium chloride (10 mM) in HepG2 (**A**), HUH7 (**B**), and mHCC (**C**) cells. Data are shown as mean ± SD (*n* = 7–9). Scale bar: 1,000 μm. (**D** and **E**) CD44 mRNA expression in HepG2 (**D**) and HUH7 (**E**) cells with and without ammonium chloride (10 mM). Data are shown as mean ± SD (*n* = 3). (**F**) CD44 surface expression by flow cytometry in control and ammonium chloride–treated (10 mM) mHCC hepatospheres. Mean fluorescence intensity (MFI) fold change ± SD (*n* = 3). (**G**–**I**) ALDH activity in HepG2 (**G**), HUH7 (**H**), and mHCC (**I**) hepatospheres with and without ammonium chloride (10 mM) (*n* = 3). *P ≤ 0.05, **P ≤ 0.005, ***P ≤ 0.0005 by 2-tailed *t* test.

Given our observation that elevated ammonia is associated with a higher HCC incidence and diminished OS, we hypothesized that ammonia may regulate CSCs because they are responsible for tumor initiation and rely on a unique microenvironment to sustain their function (20). Three-dimensional sphere culture enriches a population of cells with self-renewal properties and is one method to quantify CSCs in vitro (28). To initially test this hypothesis, we evaluated whether ammonia influenced establishment of hepatospheres. We treated the human HCC cell line HepG2 with 10 mM ammonium chloride because that is within the physiological concentration of ammonia in the liver, the concentration at which we observed peak hepatosphere formation in HepG2 cells (Supplemental Figure 3A), and has been used in prior studies studying ammonia in cancer (16). We observed that ammonium chloride treatment increased hepatosphere number and diameter in HepG2 cells (Figure 2A). To extend this finding, we next evaluated a second human HCC cell line, HUH7. Again, ammonium chloride treatment increased hepatosphere number and diameter (Figure 2B). Finally, we utilized a murine HCC cell line derived from tumors generated by overexpression of Myc and KO of p53 as previously described (hereafter termed murine HCC [mHCC]) (29). Indeed, we observed that ammonium chloride treatment increased hepatosphere number and diameter in mHCC cells (Figure 2C). These data provide evidence that ammonia promotes hepatosphere formation and growth.

CD44 is a well-established marker of CSCs in HCC and other systems (30–33). Ammonium chloride treatment increased CD44 mRNA expression in HepG2 (Figure 2D) and HUH7 hepatospheres (Figure 2E). Ammonium chloride treatment also increased CD44 surface expression in mHCC hepatospheres (Figure 2F and Supplemental Figure 3B). The enzymatic activity of aldehyde dehydrogenase (ALDH) is elevated in HCC CSCs and is another method for quantifying stemness in vitro (34, 35). Ammonium chloride treatment increased ALDH activity in HepG2 (Figure 2G), HUH7 (Figure 2H), and mHCC (Figure 2I) hepatospheres. These data suggest that ammonia promotes cancer stemness in HCC in vitro.

**Figure 3:**
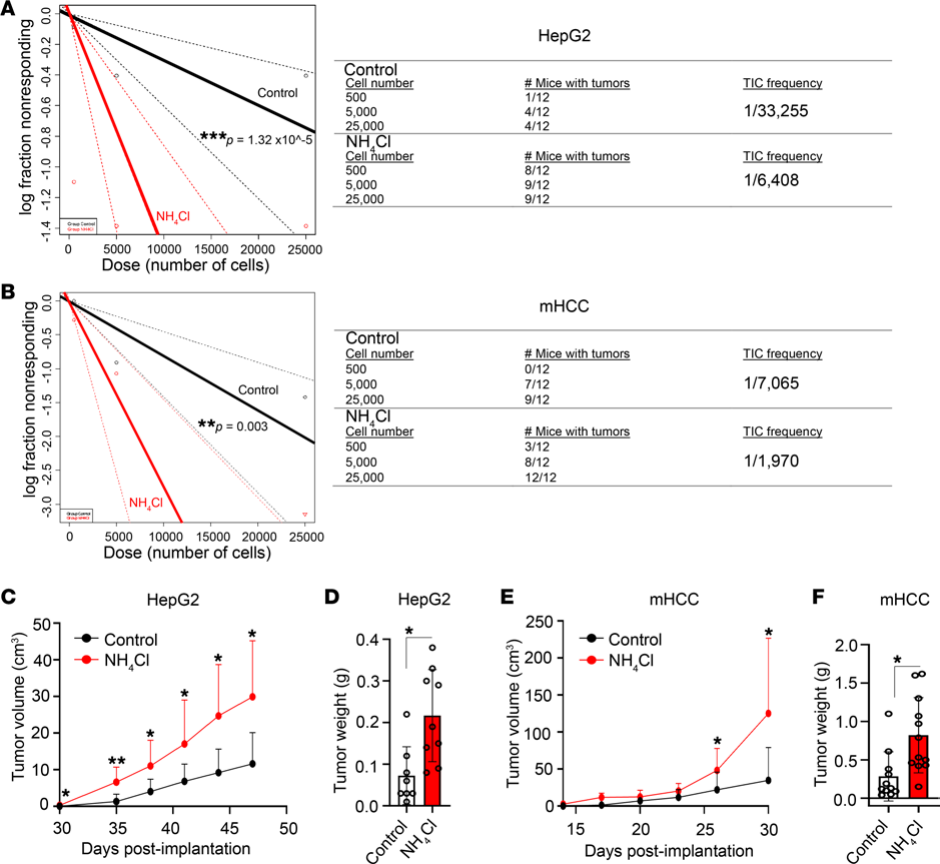
Ammonia contributes to tumor initiation in vivo. (**A** and **B**) Control and ammonium chloride–treated (10 mM) hepatospheres derived from HepG2 (**A**) and mHCC (**B**) cells were dissociated into single cells and implanted into NSG mice at the indicated cell numbers (*n* = 12 tumors per arm). Tumor initiation and TIC frequency was quantified using extreme limiting dilution analysis. (**C** and **D**) HepG2 tumor volume and tumor weight was quantified in the 500-cell titration group. (**E** and **F**) mHCC tumor volume and tumor weight was quantified in the 500-cell titration group. Data are represented as means ± SD. *P ≤ 0.05, **P ≤ 0.005, ***P ≤ 0.0005 by 2-tailed *t* test.

Limiting dilution analysis is the gold-standard method for quantifying tumor initiation in vivo (36, 37). We therefore treated HepG2 hepatospheres with ammonium chloride and conducted limiting dilution tumor initiation studies in NOD *scid* gamma (NSG) mice. We observed that ammonium chloride treatment increased the frequency of tumor-initiating cells (TICs) (Figure 3A). We next repeated limiting dilution analysis with mHCC cells. Again, ammonium chloride treatment increased the frequency of TICs (Figure 3B). We also observed that ex vivo ammonium chloride treatment of HepG2 hepatospheres prior to inoculation in NSG mice increased tumor volume (Figure 3C and Supplemental Figure 4A) and tumor weight (Figure 3D and Supplemental Figure 4B). Similarly, ex vivo ammonium chloride treatment of mHCC hepatospheres prior to inoculation in NSG mice increased tumor volume (Figure 3E and Supplemental Figure 4C) and tumor weight (Figure 3F and Supplemental Figure 4D). These data indicate that ammonia increases tumor initiation and growth in HCC in vivo.

### SLC4A11 is upregulated in HCC stem cells and functions as an ammonia importer.

**Figure 4:**
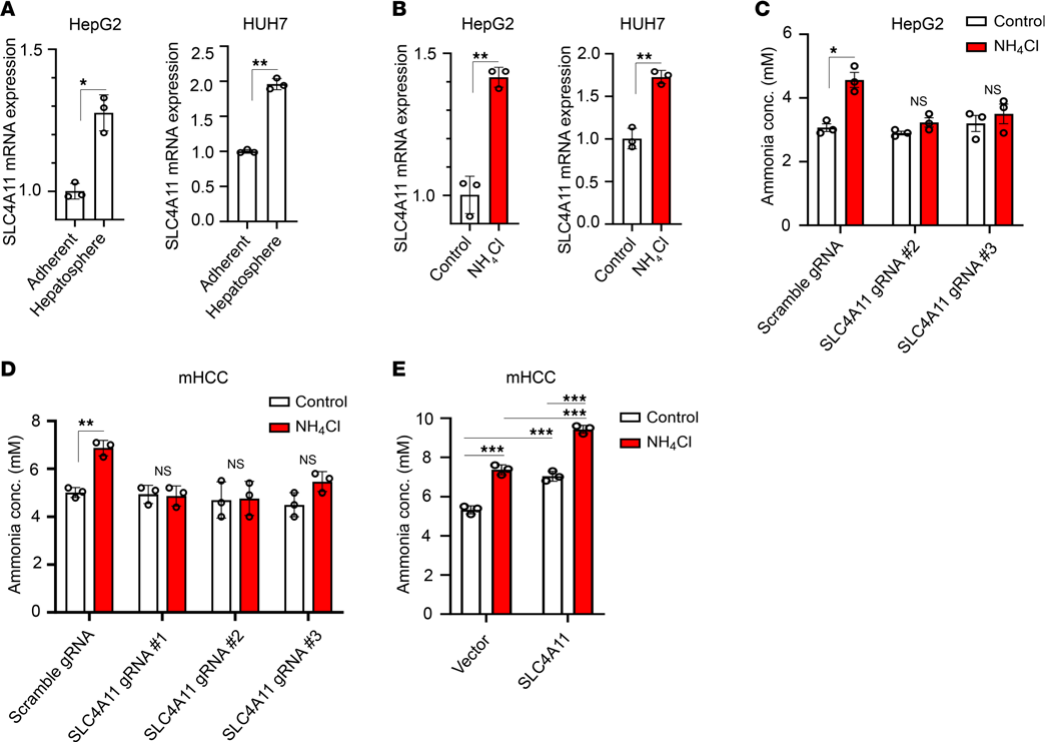
SLC4A11 functions as an ammonia importer in hepatocellular carcinoma stem cells. (**A**) SLC4A11 mRNA expression was quantified in 2D versus 3D culture of HepG2 (left) and HUH7 (right) cells. Data are shown as mean ± SD (*n* = 3). (**B**) SLC4A11 mRNA expression was quantified in HepG2 (left) and HUH7 (right) hepatospheres with and without ammonium chloride (10 mM). Data are shown as mean ± SD (*n* = 3). (**C** and **D**) SLC4A11 was depleted in HepG2 (**C**) and mHCC (**D**) cells by Crispr/Cas9 using 2–3 independent gRNAs, and ammonia concentration in control and SLC4A11-KO hepatospheres with and without ammonium chloride (10 mM) was quantified. Data are shown as mean ± SD (*n* = 3). *P ≤ 0.05, **P ≤ 0.005 by 2-tailed *t* test (**A**–**D**). (**E**) tdTomato-tagged SLC4A11 was overexpressed in mHCC cells and ammonia concentration in control, and SLC4A11 overexpression hepatospheres with and without ammonium chloride (10 mM) was quantified. Data are shown as mean ± SD (*n* = 3). ***P ≤ 0.0005 by 1-way ANOVA for the entire group with multiple comparisons using Tukey’s test.

Next, we aimed to determine the mechanism by which ammonia promotes stemness and tumor initiation. A recent report demonstrated that the ammonia transporter SLC4A11 promotes HCC growth and is associated with poor prognosis in patients (38). We therefore hypothesized that the transport of ammonia may have important functional consequences on influencing the behavior of CSCs. Indeed, SLC4A11 mRNA expression was upregulated in HepG2 and HUH7 hepatospheres when compared with adherent cells (Figure 4A) and was further induced following ammonium chloride treatment in HepG2 and HUH7 hepatospheres (Figure 4B).

SLC4A11 is known to transport ammonia both intra- and extracellularly (38–41). To determine the directionality of ammonia transport in HCC CSCs, we used Crispr/Cas9 to knock out SLC4A11 in HepG2 and mHCC cells. We designed 3 candidate human and murine gRNAs and proceeded with experiments with gRNAs that generated complete KO of SLC4A11 (human gRNAs #2 and #3 and murine gRNAs #1–#3) (Supplemental Figure 5, A–D). We observed an increase in intracellular ammonia concentration in control HepG2 and mHCC hepatospheres treated with ammonium chloride, but we did not observe an increase in SLC4A11-KO cells (Figure 4, C and D). Moreover, overexpression of SLC4A11 resulted in an increase in intracellular ammonia concentration in mHCC hepatospheres (Figure 4E and Supplemental Figure 5E). These data suggest that SLC4A11 functions as an ammonia importer in HCC CSCs.

**Figure 5:**
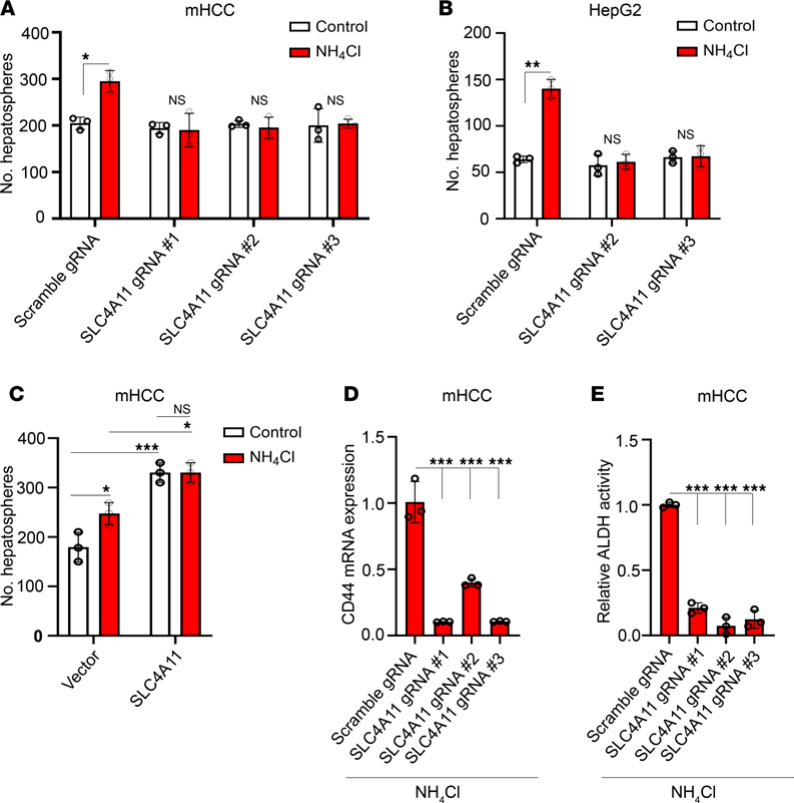
SLC4A11-mediated ammonia transport sustains a cancer stem cell phenotype. (**A** and **B**) Hepatosphere number in control and SLC4A11 KO mHCC (**A**) and HepG2 (**B**) cells with and without ammonium chloride (10 mM). Data are shown as mean ± SD (*n* = 3). *P ≤ 0.05, **P ≤ 0.005 by 2-tailed *t* test (**A** and **B**). (**C**) Hepatosphere number in control and SLC4A11 overexpressing mHCC cells with and without ammonium chloride (10 mM). Data are shown as mean ± SD (*n* = 3). (**D**) CD44 mRNA expression and (**E**) ALDH activity in control and SLC4A11 KO mHCC hepatospheres in the presence of ammonium chloride (10 mM). Data are shown as mean ± SD (*n* = 3). *P ≤ 0.05, **P ≤ 0.005, ***P ≤ 0.0005 by 1-way ANOVA with multiple comparisons using Tukey’s test (**C**–**E**).

To further assess the potential contribution of SLC4A11-mediated ammonia import in inducing stemness, we quantified hepatosphere number in control and SLC4A11-KO mHCC hepatospheres. Ammonium chloride treatment increased hepatosphere number in control cells, but it did not in SLC4A11-KO cells (Figure 5A). Similar results were obtained in HepG2 cells (Figure 5B). Furthermore, overexpression of SLC4A11 in mHCC cells resulted in an increase in hepatosphere number (Figure 5C). To provide additional evidence of a causal role of SLC4A11 in promoting stemness, we observed a reduction in CD44 mRNA expression (Figure 5D) and ALDH activity (Figure 5E) in SLC4A11-KO mHCC hepatospheres in the presence of ammonium chloride.

### Ammonia augments amino acid and nucleotide biosynthesis in a SLC4A11-dependent manner.

**Figure 6:**
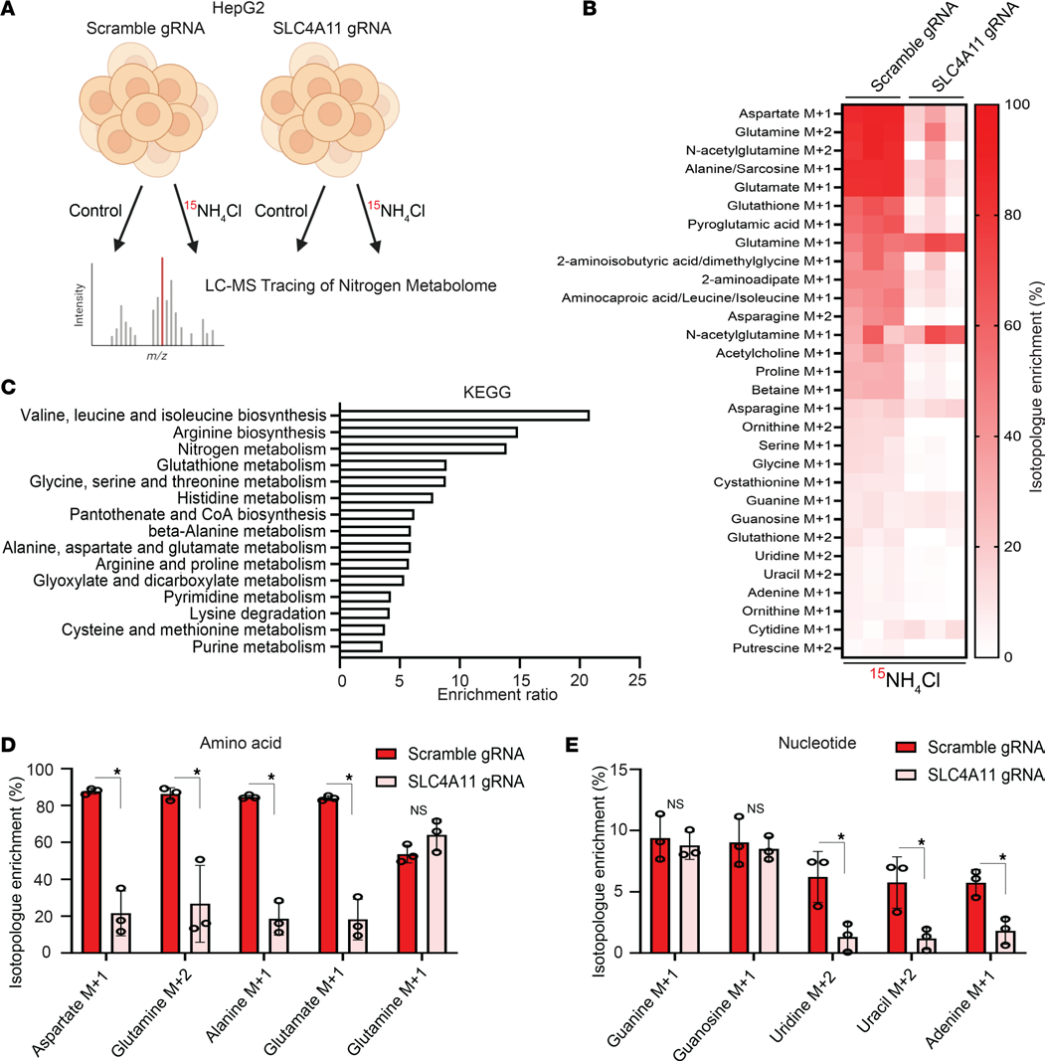
Ammonia augments amino acid and nucleotide biosynthesis in a SLC4A1-dependent manner. (**A**) Schematic depicting LC-MS–based nitrogen tracing in control and SLC4A11-KO HepG2 hepatospheres with and without 8 hours of ammonium chloride (10 mM) (created with BioRender.com). (**B**) Isotopologue enrichment of top 30 metabolites in control and SLC4A11-KO HepG2 hepatospheres (background subtracted from ^15^NH_4_Cl-treated samples, *n* = 3). (**C**) KEGG gene set enrichment analysis for 15 pathways significantly enriched by hypergeometric testing (*n* = 3). (**D** and **E**) Normalized isotopologue enrichment of representative amino acids and nucleotides in control and SLC4A11-KO HepG2 hepatospheres. *P ≤ 0.05 by 2-tailed *t* test.

An important question arising from our data is the fate of ammonia-derived nitrogen in HCC CSCs. Since we established that SLC4A11 mediates intracellular ammonia transport in CSCs, we hypothesized that ammonia incorporates into metabolic pathways that contribute to tumor growth and that this effect is diminished with SLC4A11 depletion. We therefore performed unbiased tracing of the nitrogen metabolome using high-performance liquid chromatography–mass spectrometry (HPLC-MS) by characterizing a panel of 215 isotopologues of nitrogen as previously described (15). In these experiments, control and SLC4A11-KO HepG2 hepatospheres were randomized to ± 8 hours of ^15^NH_4_Cl treatment, and the fate of ^15^NH_4_Cl was assessed by HPLC-MS in each condition (Figure 6A). In control HepG2 hepatospheres, this analysis found that ammonia-derived nitrogen primarily enters central biosynthetic pathways of amino acids and nucleotides (Figure 6B, Supplemental Figure 6A, and Supplemental Table 1). Using metabolite set enrichment analysis (MSEA), we identified 15 pathways by the Kyoto Encyclopedia of Genes and Genomes (KEGG; https://www.genome.jp/kegg/) and 14 pathways by the Small Molecule Pathway Database (SMPDB; https://www.smpdb.ca/) that were significantly enriched with ^15^NH_4_Cl treatment in control HepG2 hepatospheres (Figure 6C and Supplemental Figure 6B). We observed that mRNA expression of the enzymes glutamate dehydrogenase 1 (GDH1) and GDH2 and glutamine synthetase (GLUL), which catalyze the initial steps in ammonia assimilation, were not substantially changed in the presence of ammonium chloride and with SLC4A11 KO (Supplemental Figure 6, C–E). Isotopologues of the amino acids aspartate, glutamine, alanine, and glutamate and the nucleotides guanine, guanosine, uridine, uracil, and adenine were of the most enriched metabolites (Figure 6, D and E). In SLC4A11-KO HepG2 hepatospheres, ^15^N labeling was significantly reduced compared with controls, further substantiating our conclusion that SLC4A11 serves as a critical intracellular ammonia transporter in HCC CSCs (Figure 6, B, D, and E). Together, these data suggest that ammonia-derived nitrogen is incorporated into glutamine that serves as an intermediary for the synthesis of other amino acids and nucleotides and is reduced with SLC4A11 KO.

### Ammonia clearance reduces tumorigenesis in vivo.

**Figure 7:**
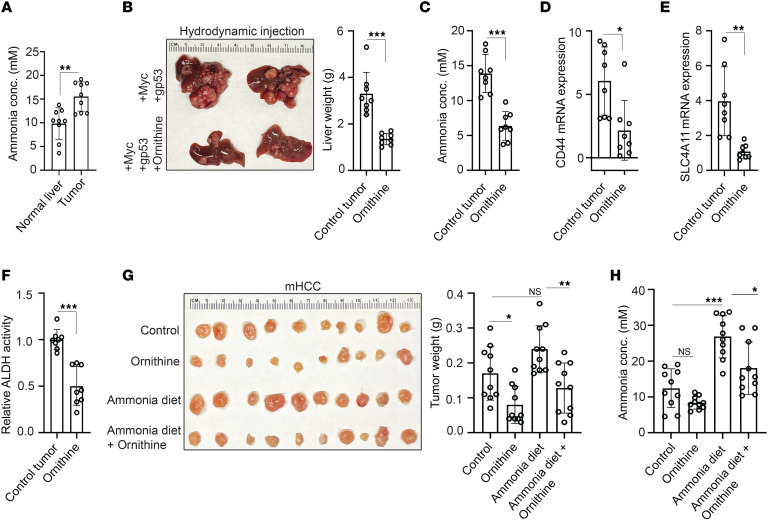
Ammonia clearance reduces tumor burden and cancer stem cell markers in vivo. (**A**) Ammonia concentration in livers of control and HCC tumor–bearing C57BL/6J mice following hydrodynamic transfection of Myc, gp53/Cas9, and sleeping beauty transposase. Data are shown as mean ± SD (*n* = 9 per arm). (**B**–**F**) Representative photo and liver weight (**B**), ammonia concentration (**C**), CD44 mRNA expression (**D**), SLC4A11 mRNA expression (**E**), and ALDH activity (**F**) of tumor-bearing C57BL/6J mice generated by hydrodynamic tail vein injection with and without ornithine treatment. Data are shown as mean ± SD (*n* = 8 per arm). *P ≤ 0.05, **P ≤ 0.005, ***P ≤ 0.0005 by 2-tailed *t* test (**A**–**F**). (**G** and **H**) Representative photo and tumor weight (**G**), and ammonia concentration (**H**) of mHCC tumors established in NSG mice with the indicated conditions. Data are shown as mean ± SD (*n* = 10 per arm). *P ≤ 0.05, **P ≤ 0.005, ***P ≤ 0.0005 by 1-way ANOVA with multiple comparisons using Tukey’s test.

We established that ammonia promotes tumor initiation and growth in HCC by affecting the function of CSCs. Using a spontaneous HCC model generated by hydrodynamic transfection of Myc, gp53/Cas9, and sleeping beauty transposase for stable genomic integration in C57BL/6J mice as previously described (29), we observed that murine livers with HCC have elevated ammonia concentrations in tumors when compared with normal control livers (Figure 7A). Ornithine is an amino acid that augments urea cycle flux to promote ammonia clearance and is used clinically to treat patients with cirrhosis (42); we have previously shown that ornithine can lower intratumoral ammonia within the liver (14). We therefore tested the effects of ammonia clearance using ornithine in mice with HCCs generated by hydrodynamic transfection and observed a striking reduction in tumor growth as measured by liver weight (Figure 7B). Ornithine treatment at physiologically relevant doses also reduced intratumoral ammonia concentration (Figure 7C), CD44 mRNA expression (Figure 7D), SLC4A11 mRNA expression (Figure 7E), and ALDH activity (Figure 7F). To further test the effects of ammonia reduction on tumor growth, we established mHCC xenografts and treated mice fed a standard diet with ornithine, fed mice a high-ammonia diet, and treated mice fed a high-ammonia diet with ornithine. In this analysis, we found that ornithine treatment in mice fed a high-ammonia diet resulted in smaller tumor weights and lower intratumoral ammonia concentrations (Figure 7, G and H). These experiments demonstrate that ammonia clearance reduces CSC properties and tumor growth in vivo.

## Discussion

The results of this study demonstrate that ammonia contributes to the function of CSCs and promotes tumor initiation in HCC. In preclinical models, the transporter SLC4A11 sustained high levels of intracellular ammonia and is at the nexus of a network that rewired the nitrogen metabolome to promote amino acid and nucleotide biosynthesis in HCC CSCs. In patients with cirrhosis, elevated ammonia was associated with higher HCC incidence when correcting for liver function and using propensity score matching. Additionally, in patients with HCC, elevated ammonia was associated with worse OS. These findings increase our understanding of the role of ammonia in tumorigenesis. They also have substantial clinical implications because multiple pharmacologic agents that are commonly used to promote ammonia clearance in patients with cirrhosis at ranges established to have clinical impact could be repurposed to be tested as therapeutic or preventative agents in trials.

A major finding of this study is that ammonia contributes to tumor initiation and growth in HCC by regulating CSC function. This observation is important as dysregulated ammonia metabolism is a common clinical finding in patients with cirrhosis, which is the most significant risk factor in developing HCC (43). While other studies have demonstrated causal roles of ammonia in promoting the growth of other malignancies (14, 15), our finding of ammonia conferring CSC properties has not been previously reported. Resisting oxidative damage is a hallmark of CSCs that contributes to their long-term self-renewal potential (44) as well as therapy resistance (45). Therefore, anabolic pathways fueled by ammonia-derived nitrogen may have both a direct role in promoting tumor growth and an indirect role by helping to maintain low levels of baseline reactive oxygen species.

Our data build on the role of amino acid and nucleotide biosynthesis as drivers of tumorigenesis and directly implicate glutamine-mediated metabolic pathways in contributing to HCC CSC function (46). Targeting CSCs has proven to be a challenge due to some redundancy with other cell populations, but exploiting their distinct metabolic requirements holds potential in the development of new therapeutic applications (44). Several inhibitors of glutamine and related pathways exist that may be explored to address therapy resistance in HCC in subsequent work (47). Given the multifaceted role of glutamine metabolites in biosynthetic pathways, redox balance, cell signaling, and gene expression, our data provide a mechanistic understanding of how HCC CSCs may adapt to their microenvironment to promote tumor propagation.

We demonstrated that SLC4A11 is upregulated in HCC CSCs where it functions as an ammonia importer, consistent with prior studies evaluating the mode of transport of SLC4A11 (41). While much of the literature has focused on SLC4A11 mutations and their contribution to congenital hereditary endothelial dystrophy (48, 49), recent findings implicate SLC4A11 in HCC by a mechanism that involves ammonia excretion to resist senescence (38). Although the directionality of ammonia transport may be system or cell type dependent, our conclusions are substantiated by our unbiased tracing of the nitrogen metabolome that demonstrated reduced intracellular nitrogen incorporation in SLC4A11-depleted cells. Our data are consistent in the broader view that SLC4A11 is an important oncogene in HCC. The finding that SLC4A11 confers CSC properties in HCC adds to the understanding of its role in tumorigenesis, warranting further work in HCC and other malignancies.

Dysregulation of the urea cycle has been observed in various malignancies, including HCC (50, 51). This metabolic disruption can be influenced by factors such as p53, which has been shown to regulate urea cycle enzyme expression (52). As a result, in patients with HCC, there are 2 distinct mechanisms that contribute to elevated ammonia: (a) chronic liver disease and (b) repression of urea cycle enzymes by tumor cells. Our findings that HCC CSCs assimilate ammonia into amino acid and nucleotide biosynthetic pathways indicate that this is an adaptive mechanism to facilitate tumor growth because the ability of ammonia to be detoxified into urea is reduced. The fact that ornithine, a metabolite that enhances urea cycle flux, reduced tumorigenesis in vivo highlights the potential of restoring urea cycle function as a therapeutic strategy for HCC. These results emphasize the need for further investigation into the role of the urea cycle in HCC stem cells, and this may offer additional insights into targeted treatments and mechanisms underlying tumor development.

While this is the largest report to date evaluating clinicopathologic features that correlate with HCC incidence, it remains a retrospective observational study that will require prospective validation of findings. In addition, our work reveals a previously unreported mechanism that involves ammonia-mediated regulation of the nitrogen metabolome that promotes stemness and tumor initiation in HCC. Our clinical data provide correlative evidence that elevated ammonia is associated with higher cancer incidence and poor OS in patients with HCC. Targeting ammonia as a therapeutic strategy in HCC has not been explored, and our work suggests the need for clinical trials evaluating whether suppression of ammonia would mitigate HCC incidence or severity in patients with cirrhosis.

## Methods

### Sex as a biological variable.

Male mice were used in this study, given the disproportionate incidence of HCC among males versus females (53, 54). We anticipate that the results of this study are relevant to both sexes, given its mechanistic basis that applies to HCC initiation in male and female patients.

### Mice.

Male 8- to 12-week-old C57BL/6J and NSG mice were acquired from The Jackson Laboratory. Mice were fed a standard chow diet ad libitum and housed in a pathogen-free, temperature-controlled room with a 12-hour light/dark cycle.

### Patients.

This study utilized electronic medical record data from the Veterans Health Administration (VHA) Corporate Data Warehouse (CDW), which includes data for all veterans receiving care through VHA facilities nationwide. This study was approved by the Veterans Affairs Ann Arbor Research and Development Board. We identified patients diagnosed with cirrhosis from 1999 to 2024 using inpatient/outpatient International Classification of Diseases 9/10 (ICD9/10) codes as well as Fibrosis-4 (FIB4) score > 3.25 as previously described (55). The date of cirrhosis diagnosis was defined as the index date in HCC incidence analyses (Cohort 1), and the date of HCC diagnosis was defined as the index date in OS analyses (Cohort 2). Cancer diagnoses and dates of diagnosis were identified by the VA Cancer Registry System. OS was defined as the time from the index date to death from any cause. Date of death was obtained from the VA death registry. Liver function was assessed using ALBI, eCTP, and MELD scores as previously described (25–27). Ammonia levels were obtained from the structured laboratory data. Mean ammonia serum concentration was calculated for patients with greater than 1 ammonia laboratory value in the system. For HCC incidence analyses, 3-year mean ammonia values (1 year prior to cirrhosis diagnosis and 2 years after cirrhosis diagnosis) were used. For OS analyses, 2-year mean ammonia values (1 year prior to HCC diagnosis and 1 year after HCC diagnosis) were used.

Patients were stratified into low and high ammonia groups based on ≥ 48.5 μM/L, which was determined by using the Youden Index (24). To assess the association between ammonia concentration and HCC incidence, patients with mean ammonia concentrations ≤ 100 μM/L were included in this analysis because concentrations > 100 μM/L are associated with acute liver failure and increased mortality (56, 57). Patient-level mean ammonia levels were binned every 5 μM/L, and the proportion of patients who developed HCC evaluated. Propensity score matching was performed via the Toolkit for Weighting and Analysis of Nonequivalent Groups (TWANG) package. The weights were estimated using the covariate balancing propensity score method taking into account age, sex, race, ethnicity, ALBI, eCTP, MELD, Charleson Comorbidity Index, cirrhosis etiology, and HCC screening intensity (by either magnetic resonance imaging or ultrasound). The RadBERT large language model was trained to identify the subset of patients with cirrhosis with negative HCC screens (58). Patients were censored at the date of last known follow-up, defined as the most recent encounter with a Veterans Affairs (VA) provider. Patients with ongoing follow-up past April 1, 2024, were administratively censored at that time. Demographics including race, sex, and age were obtained through the Master Patient Index. Analysis was performed with R v4.3.1 (R Core Team) and Python v3.10.4 (Python Software Foundation).

### Reagents and antibodies.

[^14^N]ammonium chloride (catalog 213330), [^15^N]ammonium chloride (catalog 299251), [^14^N]ammonium acetate (catalog A1542), and ornithine monohydrochloride (catalog O6503) were purchased from MilliporeSigma. Immunoblotting antibodies were acquired as follows: SLC4A11 (PA5-101889, Thermo Fisher Scientific), RFP that cross-reacts with tdTomato (ab124754, Abcam), and actin (3700, Cell Signaling Technology). CD44 antibody used for flow cytometry was acquired from Thermo Fisher Scientific (catalog 24-0551-82).

### Cell culture.

HepG2 cells were provided by Weiping Zou (University of Michigan) and cultured in Eagle’s Minimum Essential Medium with 10% FBS. HUH7 cells were provided by Susan Uprichard (Loyola University, Chicago, Illinois, USA) and cultured in DMEM (MilliporeSigma) with 10% FBS (Thermo Fisher Scientific). mHCC cells were generated from a C57BL/6J mouse bearing HCCs generated by hydrodynamic transfection of Myc, gp53/Cas9, and sleeping beauty transposase (as described below) and were provided by Viraj Sanghvi (Columbia University, New York, New York, USA) (29). mHCC cells were cultured in DMEM with 10% FBS. All cell lines were screened for mycoplasma at least every other week and tested negative before use.

### Constructs and Crispr/Cas9 KO studies.

CHOPCHOP (https://chopchop.cbu.uib.no/) was used to design gRNAs for KO of SLC4A11 in human and murine cell lines. VectorBuilder (https://en.vectorbuilder.com/) was used to clone gRNA sequences into pLV lentiviral vectors with a puromycin resistance cassette. The following gRNA sequences were used: human SLC4A11 #1, 5′-AAGGCGATATCCGAGAACA-3′ (VectorBuilder ID: VB231017-1759pac); human SLC4A11 #2, 5′-TCGCAGAATGGATACTTCG-3′ (VectorBuilder ID: VB231017-1760qhc); human SLC4A11 #3, 5′-GTCCGCAGCACGTTATCCA-3′ (VectorBuilder ID: VB231017-1761uzc); murine SLC4A11 #1, 5′-ATTCCAATCCGGTATGACA-3′ (VectorBuilder ID: VB231017-1184qmw); murine SLC4A11 #2, 5′-TACTGCACCCCTCGGACAG-3′ (VectorBuilder ID: VB231018-1344xta); murine SLC4A11 #3, 5′-GTCCGTGCACACCGGGACC-3′ (VectorBuilder ID: VB231017-1758mtd); and scramble gRNA, 5′-TGTAGTTCGACCATTCGTG-3′ (VectorBuilder ID: VB231017-1185cny). Streptococcus pyogenes Cas9-high fidelity variant 1 (SpCas9-HF1) was cloned into a pLV lentiviral vector with a hygromycin resistance cassette (VectorBuilder ID: VB231012-1059dhf) (59). Cells were cotransduced with a gRNA and SpCas9-HF1 and antibiotic selected to generate stable KO lines. Murine SLC4A11 was connected to tdTomato by a 3× GGS linker at its C-terminus and used for overexpression studies (VectorBuilder ID: VB231018-1359kbx). Plasmids used for hydrodynamic transfection (Myc, gp53/Cas9, and sleeping beauty transposase) were provided by Viraj Sanghvi and have been previously described (29).

### Hepatosphere assay.

Approximately 2,000 cells were plated in ultra-low attachment 6-well plates (Corning, catalog 3471) in triplicate in advanced DMEM/F12 supplemented with 20 ng/mL human recombinant Epidermal Growth Factor (Stem Cell Technologies, catalog 78136), 20 ng/mL human recombinant Fibroblast Growth Factor (Stem Cell Technologies, catalog 78134), and 0.2% B27 (Thermo Fisher Scientific, catalog 17504044). Hepatosphere number was quantified when spheres reached at least 100 μM diameter, and representative bright-field micrographs were captured using a BioTek BioSpa 8 automated incubator. ImageJ (NIH; https://imagej.net/ij/) was used to quantify hepatosphere diameter in bright-field micrographs.

### Quantitative PCR.

RNA was extracted using an RNA isolation kit (Qiagen, catalog 74134) and cDNA was produced using high-capacity cDNA reverse transcription kit (Thermo Fisher Scientific, catalog 4368814). SYBR green was used as the master mix (Thermo Fisher Scientific, catalog A25742). Experiments were normalized to GAPDH and performed in triplicate. The following human primer sequences were used: GAPDH, forward 5′-GGAGCGAGATCCCTCCAAAAT-3′, reverse 5-GGCTGTTGTCATACTTCTCATGG-3′; SLC4A11, forward 5′-ATGTCGCAGAATGGATACTTCG-3′, reverse 5′-AAAAACGGATACTCTCGCCAG-3′; CD44, forward 5′-CTGCCGCTTTGCAGGTGTA-3′, reverse 5′-CATTGTGGGCAAGGTGCTATT-3′; GLUD1, forward 5′-CCTGGGCGAAGCGCTGTTGCT-3′, reverse 5′-GGGCTGTCCCCGGGCCCA-3′ (15); GLUD2, forward 5′-TGGCCAAAGCGCTGCTGCC-3′, reverse, 5′-GCTGTCCGCGGCCCCG-3′ (15); and GLUL, forward 5′-AAGAGTTGCCTGAGTGGAATTTC-3′, reverse, 5′-AGCTTGTTAGGGTCCTTACGG-3′. The following murine primer sequences were used: GAPDH, forward 5′-TGGCCTTCCGTGTTCCTAC-3′, reverse 5′-GAGTTGCTGTTGAAGTCGCA-3′; SLC4A11 forward 5′-CAGGACTCCGGTGAATACTTCT-3′, reverse 5′-GATGCTCTCGCCAGACACAA-3′; and CD44 forward 5′-TCGATTTGAATGTAACCTGCCG-3′, reverse 5′- CAGTCCGGGAGATACTGTAGC-3′.

### ALDH activity assay.

Samples were processed for ALDH activity using a colorimetric kit (MilliporeSigma, catalog MAK082). All experiments were performed in triplicate and normalized to the control condition to quantify relative changes in ALDH activity.

### Flow cytometry.

Hepatospheres were dissociated into single cells using an enzyme-free dissociation reagent (STEMCELL Technologies, catalog 100-0485), processed for CD44 flow cytometry using a Fortessa equipped with 4 lasers (BD Biosciences), and analyzed using FlowJo software (https://www.flowjo.com/).

### Immunoblotting.

Cells were washed in 1× phosphate-buffered saline and lysed using radioimmunoprecipitation assay buffer (Thermo Fisher Scientific, catalog 89900) supplemented with protease (MilliporeSigma, catalog 11836153001) and phosphatase (Thermo Fisher Scientific, catalog A32957) inhibitors. Laemmli 4× sample buffer (Bio-Rad, catalog 1610747) was added to each sample. The protein lysate was subsequently boiled for 10 minutes at 100°C and separated using SDS-PAGE.

### Ammonia quantification.

Samples were deproteinated using a methanol-chloroform-water gradient as previously described (14). An ammonia assay kit (MilliporeSigma, catalog MAK310) was used to quantify ammonia concentration in cells or tissues.

### Nitrogen tracing and MS.

Hepatospheres were generated from HepG2 cells expressing either a scramble gRNA or SLC4A11 gRNA #2. Hepatospheres plates were randomized to control versus ^15^NH_4_Cl treatment (10 mM). After 8 hours of ^15^NH_4_Cl treatment, cells were harvested and pellets were frozen in –80°C. After conducting 3 independent biological replicates, frozen pellets were processed for HPLC-MS.

For signal normalization, an internal standard (IS) solution containing valine-d_8_, creatinine-d_3_, glutamine-d_5_, phenylalanine-^13^C_6_, and isoleucine-d_10_ (100 μg/mL) was prepared in water/methanol, 1:1 (v/v). Sample extraction was accomplished by adding 40–60 μL IS solution followed by 750–1,000 μL water/methanol, 2:8 (v/v). Protein was precipitated by sonication and centrifugation (15,000*g*), and samples were transferred to an HPLC autosampler vial and injected directly for LC-MS analysis. Data analysis was performed using Xcalibur Quan Browse software (Thermo Fisher Scientific; version 4.4.16.14). A custom processing method was created containing all compounds and their isotopologues as previously described (15). A mass accuracy filter of 5 ppm was utilized. Extracted ion chromatograms resulting from the exact *m/z* values were generated for acidic and basic conditions. Peaks were manually reviewed in both polarities for all samples run in both mobile phases. Valine-d_8_ (Cayman Chemical) was used as the IS, and the positive-ion data using the acidic LC-MS method were used as these generated reliable peak shapes for consistent integration. Data were analyzed using the following formula: area ratio analyte/internal standard (× 1,000). This value was used to calculate isotopologue enrichment (percentage) by quantifying the ratio of the analyte to that of the analyte and its M+0 isotopologue. Respective untreated controls were subtracted from each condition. In some cases, analytes sharing the exact same *m/z* value were not resolved chromatographically and could not be integrated separately. These analytes are reported in the same rows in the respective figures.

### MSEA.

MSEA was performed using MetaboAnalyst 6.0 (https://www.metaboanalyst.ca/). Compounds with ^15^N labeling were analyzed by the KEGG and SMPDB databases, and significantly enriched pathways by hypergeometric testing are depicted.

### Limiting dilution analysis.

Control and ammonia-treated hepatospheres were dissociated into single cells using an enzyme-free dissociation reagent (STEMCELL Technologies, catalog 100-0485), counted, and implanted s.c. with X-Vivo Serum-Free Media (Lonza, catalog 04-380Q) into NSG mice with Matrigel. Control hepatospheres were implanted on the left and ammonia-treated hepatospheres were implanted on the right to assess the effects of each condition in the same mouse. Extreme limiting dilution analysis was used to quantify the TIC frequency (36).

### Hydrodynamic and xenograft studies.

Hydrodynamic tail vein injections were performed in WT male C57BL/6J mice and have been previously described (29). Briefly, a 2 mL plasmid mix of Myc transposon (10 μg), p53 gRNA (10 μg), Cas9 (10 μg), and sleeping beauty transposase (4 μg) was injected into a single mouse. For xenograft experiments, 1 million mHCC cells were s.c. implanted in NSG mice with X-Vivo Serum-Free Media (Lonza, catalog 04-380Q) with Matrigel. Ornithine was delivered by i.p. injection in C57BL/6J mice or s.c. implantation in NSG mice at a concentration of 20 mM as previously described (14).

### Ammonium acetate diet.

Powdered chow was mixed with 25% ammonium acetate and water using a KitchenAid food mixer as previously described (14). Food pellets were generated and dried in a dehydrator for 72 hours before use.

### Statistics.

Experimental conditions were performed in triplicate, and reproducibility of each panel is indicated in the respective figure legend. Representative data are displayed in the figures unless otherwise noted. Mice were randomized to each experimental condition. Details on statistical analyses are described in the figure legends and are reported as the Data are shown as mean ± SD. Statistical significance between 2 groups was determined by 2-tailed *t* test. Means across multiple groups were compared using 1-way ANOVA, and Tukey’s test was used to perform paired comparisons. Statistical significance is described as *P* ≤ 0.05.

### Study approval.

Animal experiments were conducted in accordance with the Association for Assessment and Accreditation of Laboratory Animal Care international guidelines and approved by the IACUC at the University of Michigan.

### Data availability.

Reagents that were generated throughout this study are available from the lead contacts with a completed Materials Transfer Agreement. Values for all data points in graphs are reported in the Supporting Data Values file.

## Author contributions

ALE, MDG, and YMS designed experiments and wrote the manuscript. ALE executed experiments. MOE, JJ, ZW, ANP, EAH, and AKH provided experimental support. MG performed HPLC-MS. TSL provided expertise throughout the entirety of the project and helped write the manuscript. HNB, VRS, TLF, GLS, EBT, AWT, NR, CPC, ID, JAM, AKB, DAE, EC, JRE, KCC, TJF, DRW, MAM, DTC, and MSW provided feedback on data. All authors read and approved the final manuscript.

## Supplementary Material

Supplemental data

Unedited blot and gel images

Supporting data values

## Figures and Tables

**Table 1:**
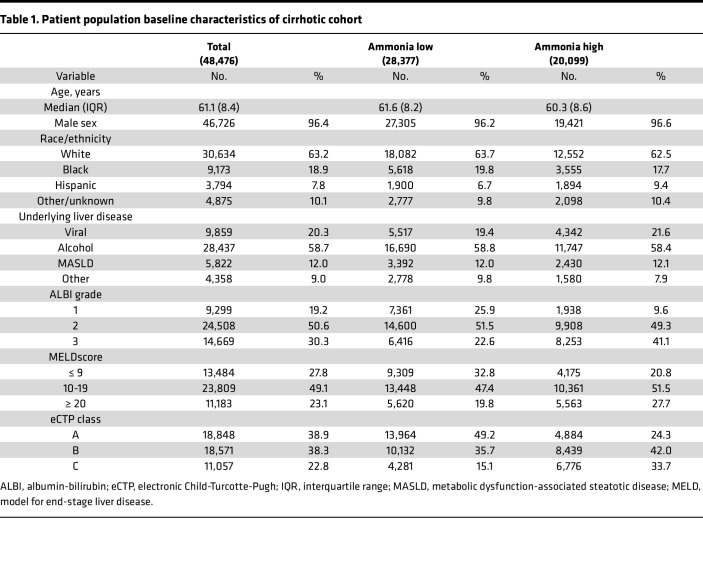
Patient population baseline characteristics of cirrhotic cohort

**Table 2:**
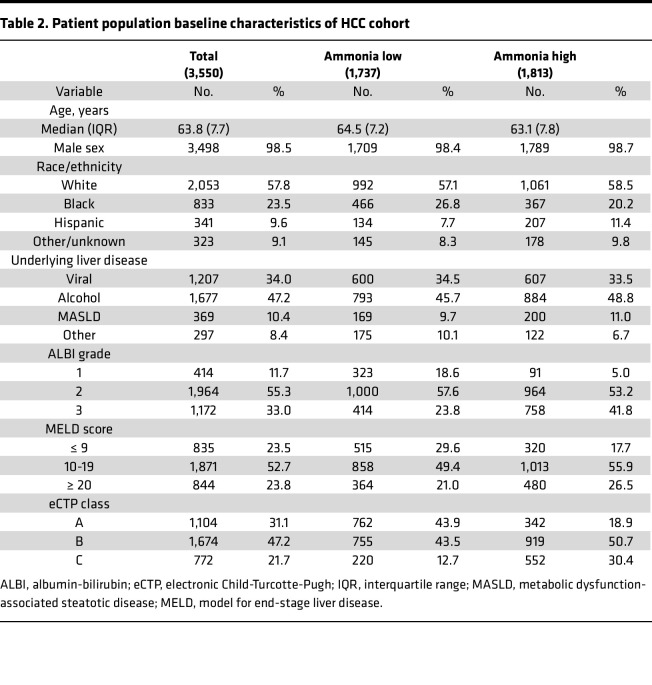
Patient population baseline characteristics of HCC cohort

## Version 1 09/17/2024

In-Press Preview

## Version 2 11/08/2024

Electronic publication
