# Supplementary material for: SLC4A11 mediates ammonia import and promotes cancer stemness in hepatocellular carcinoma
Source: JCI Insight. 2024 Nov 8;9(21):e184826. doi: 10.1172/jci.insight.184826 (PMC11601557; doi:10.1172/jci.insight.184826)
Supplement: Supplemental data [file jciinsight-9-184826-s290.pdf]

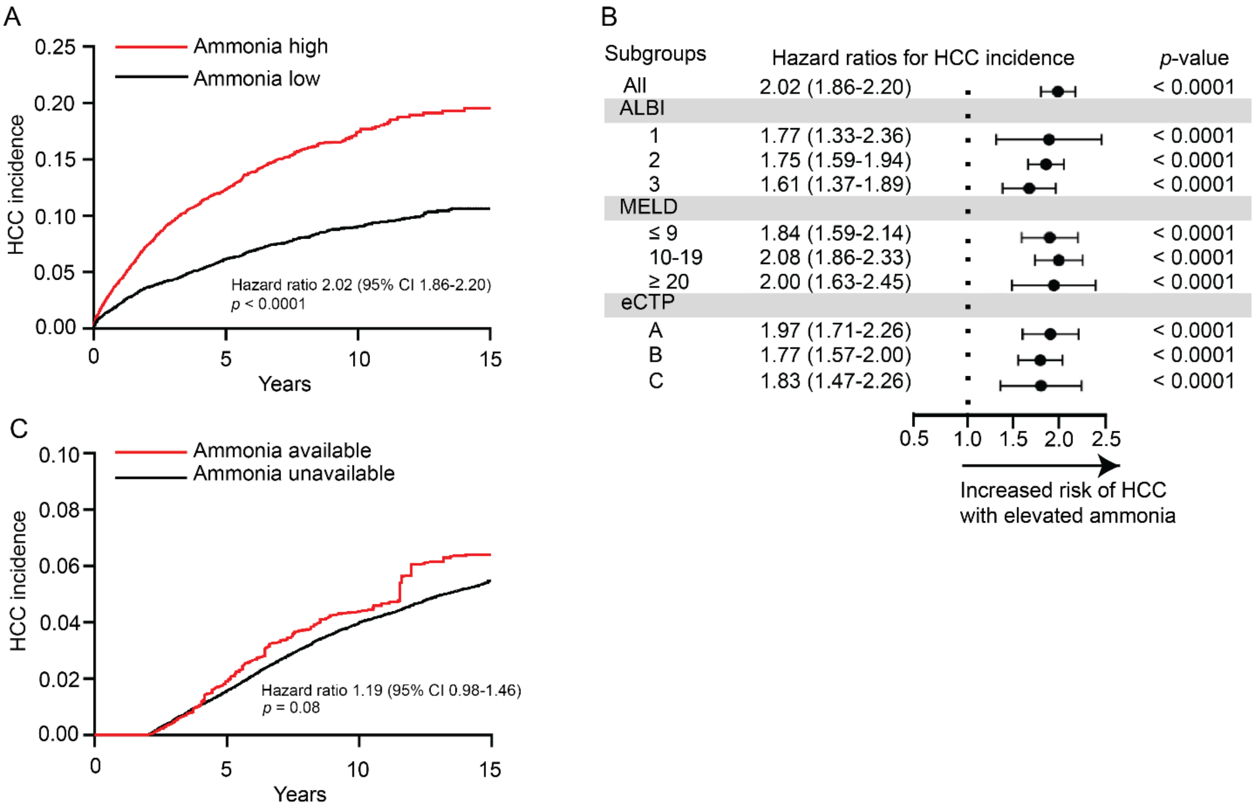

Supplemental Figure 1. (**A**) Cumulative HCC incidence stratified by mean ammonia levels (ammonia high, n = 20,099; ammonia low, n = 28,377). (**B**) HCC incidence by ALBI grade, MELD score, and eCTP class. (**C**) Two-year landmark analysis of HCC incidence in patients with available and unavailable ammonia using propensity score matching. Hazard ratio log-rank test, p values, and 95% confidence intervals indicated.

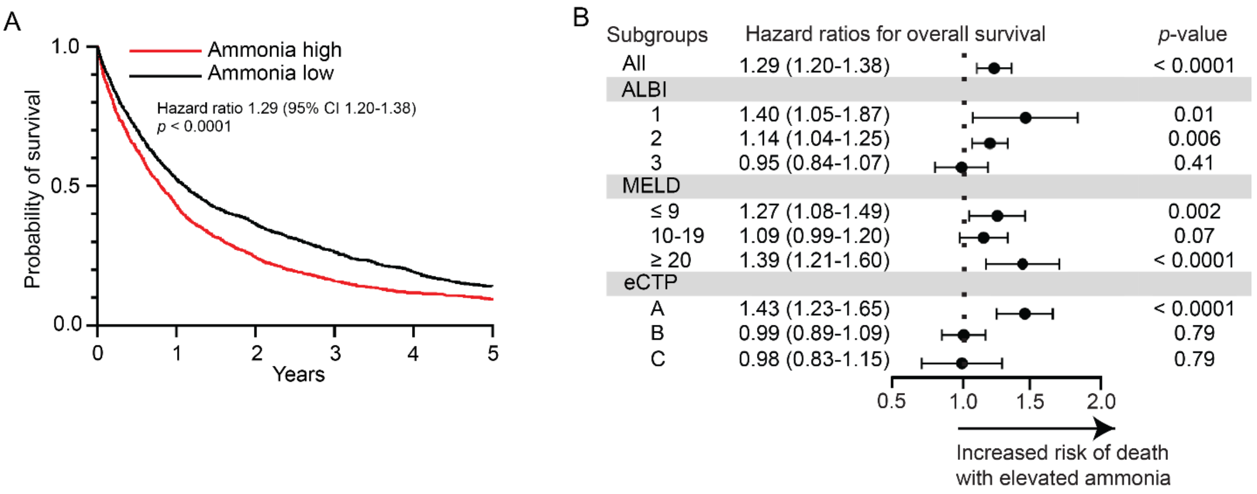

Supplemental Figure 2. (**A**) OS of patients with HCC stratified by mean ammonia levels (ammonia high, *n* = 1,813; ammonia low, *n* = 1,737). (**B**) OS of patients diagnosed with HCC by ALBI grade, MELD score, and eCTP class. Hazard ratio log-rank test, *p* values, and 95% confidence intervals indicated.

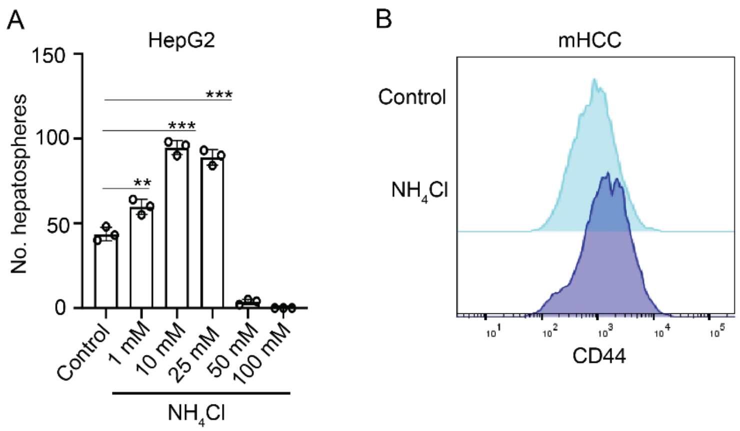

Supplemental Figure 3. (**A**) Hepatosphere number of HepG2 cells following treatment with the indicated concentrations of ammonium chloride. Mean ± SD (n = 3). ***$p \leq 0.0005$ by one-way ANOVA for entire group with multiple comparisons using Tukey's test, key comparisons are indicated in (**A**). (**B**) Histogram depicting CD44 surface expression in mHCC cells with and without ammonium chloride (10 mM).

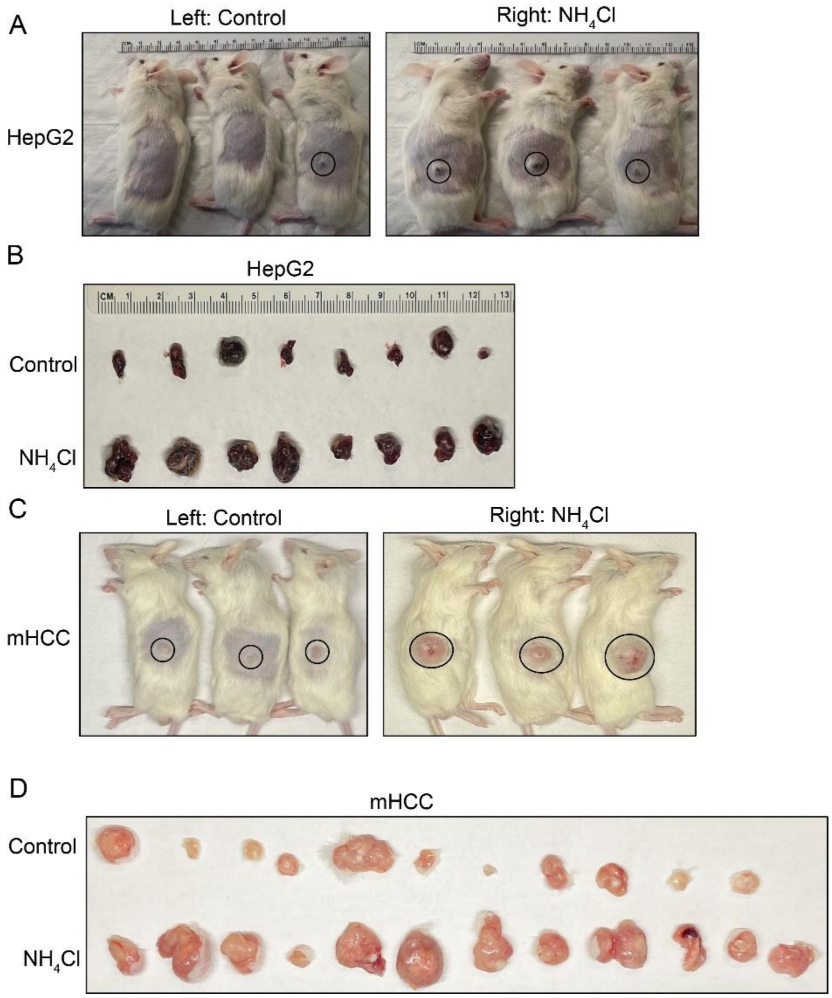

Supplemental Figure 4. (**A**) Representative photograph of HepG2 tumor bearing NSG mice with and without pre-treatment with ammonium chloride. (**B**) Representative photo of dissected HepG2 tumors implanted in NSG mice with and without pre-treatment with ammonium chloride. (**C**) Representative photograph of mHCC tumor bearing NSG mice with and without pre-treatment with ammonium chloride. **(D)** Representative photo of dissected mHCC tumors implanted in NSG mice with and without pre-treatment with ammonium chloride.

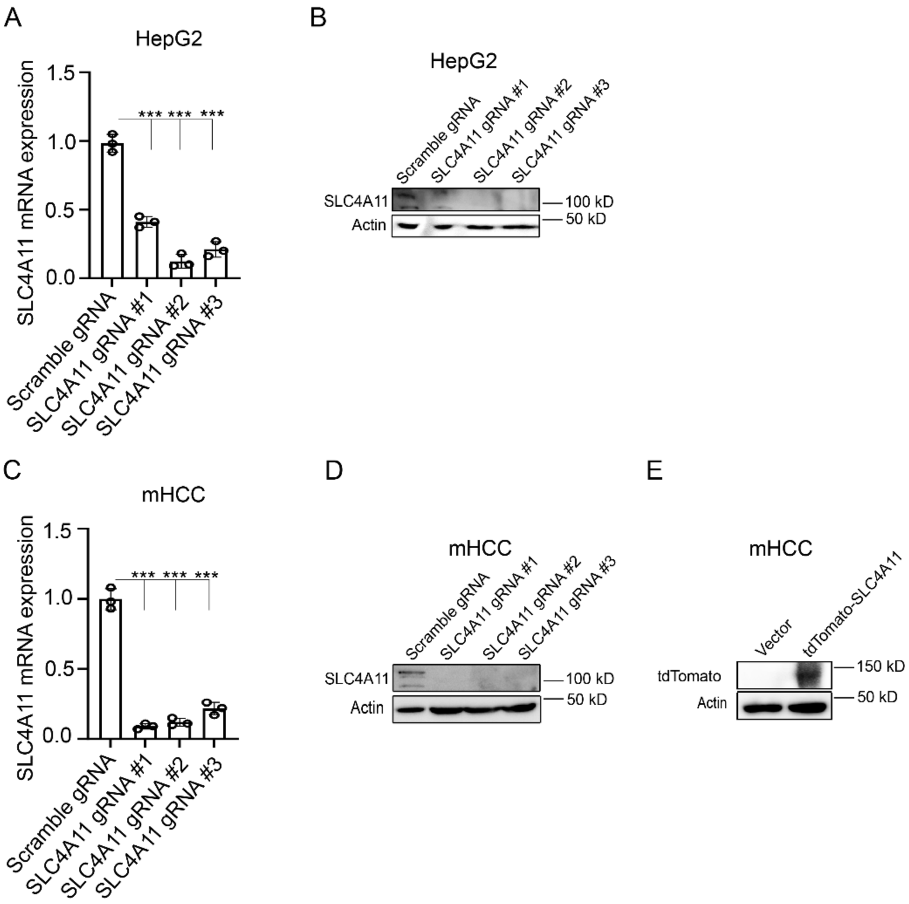

Supplemental Figure 5. SLC4A11 was depleted in HepG2 cells by Crispr/Cas9 using 3 independent gRNAs and (**A**) mRNA expression was quantified by qPCR (***$p \leq 0.0005$ by one-way ANOVA for entire group) and (**B**) protein abundance was quantified by immunoblotting in control and SLC4A11 KO cells. SLC4A11 was depleted in mHCC cells by Crispr/Cas9 using 3 independent gRNAs and (**C**) mRNA expression was quantified by qPCR (***$p \leq 0.0005$ by one-way ANOVA for entire group) and (**D**) protein abundance was quantified by immunoblotting in control and SLC4A11 KO cells. (**E**) tdTomato-tagged SLC4A11 was overexpressed in mHCC cells, which was verified by immunoblotting. ** $p \leq 0.005$, *** $p \leq 0.0005$ by one-way ANOVA with multiple comparisons using Tukey's test, key comparisons are indicated in (**A**) and (**C**).

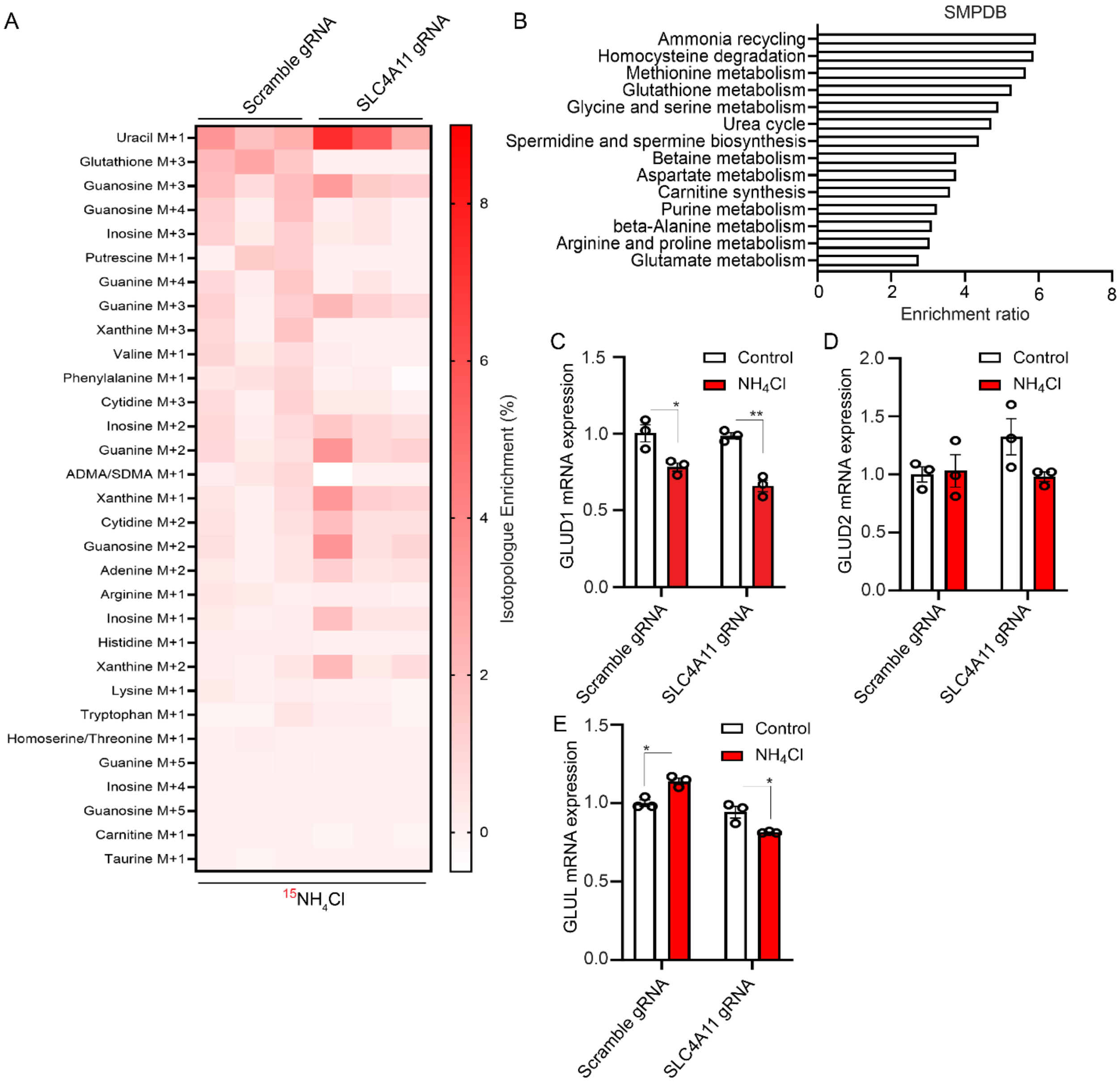

Supplemental Figure 6. (**A**) Isotopologue enrichment in control and SLC4A11 KO HepG2 hepatospheres (background subtracted from $^{15}NH_4Cl$ treated samples, *n* = 3). (**B**) MSEA pathway analysis by SMPDB of 14 pathways determined to be significantly enriched by hypergeometric testing (*n* = 3). Control and SLC4A11 hepatospheres were grown with and without ammonium chloride (10 mM) and mRNA expression of (**C**) GLUD1 (****p* ≤ 0.0005 by one-way ANOVA for entire group), (**D**) GLUD2 (*p* NS by one-way ANOVA for entire group), and (**E**) GLUL (****p* ≤ 0.0005 by one-way ANOVA for entire group) was quantified. Mean ± SD (*n* = 3). * *p* ≤ 0.05 by one-way ANOVA with multiple comparisons using Tukey's test, key comparisons are indicated in (**C**)-(**E**).

Supplementary Table 1: Metabolites not detected in $^{15}NH_4Cl$ tracing

Acetyl-glycine M+1	Dihydroorotate M+1	NAD M+2	Choline M+1
Adenine M+3	Dihydroorotate M+2	NAD M+3	Niacinamide M+1
Adenine M+4	Folate M+1	NAD M+4	Creatine/hydrolyzed phosphocreatine M+1
Adenine M+5	Folate M+2	NAD M+5	Phosphocholine M+1
Adenylosuccinate M+1	Folate M+3	NAD M+6	NAD M+1
Adenylosuccinate M+2	Folate M+4	NAD M+7	
Adenylosuccinate M+3	Folate M+5	Niacinamide M+2	
Adenylosuccinate M+4	Folate M+6	Nicotinate M+1	
Adenylosuccinate M+5	Folate M+7	NMMA M+1	
ADMA/SDMA M+2	Glucosamine M+1	NMMA M+2	
ADMA/SDMA M+3	Glycocholate M+1	NMMA M+3	
ADMA/SDMA M+4	GMP M+1	NMMA M+4	
α-glycerophosphocholine M+1	GMP M+2	p-methylhippuric acid/phenylacetylglycine M+1	
AMP M+1	GMP M+3	Pantothenate M+1	
AMP M+2	GMP M+4	Phosphocreatine dimer/Creatine M+1	
AMP M+3	GMP M+5	Phosphocreatine dimer/Creatine M+2	
AMP M+4	Guanidoacetic acid M+1	Phosphocreatine dimer/Creatine M+3	
AMP M+5	Guanidoacetic acid M+2	Phosphoethanolamine M+1	
Arginine M+2	Guanidoacetic acid M+3	Pyridoxine M+1	
Arginine M+3	Hippurate M+1	Quinolinate M+1	
Arginine M+4	Histamine M+1	Spermidine M+1	
Arginosuccinate M+1	Histamine M+2	Spermidine M+2	
Arginosuccinate M+2	Histamine M+3	Spermidine M+3	
Arginosuccinate M+3	Histidine M+2	Spermine M+1	
Arginosuccinate M+4	Histidine M+3	Spermine M+2	
Cadaverine M+1	Homocysteine M+1	Spermine M+3	
Cadaverine M+2	Homocystine M+1	Spermine M+4	
Carnosine M+1	Homocystine M+2	Thymine M+1	
Carnosine M+2	IMP M+1	Thymine M+2	
Carnosine M+3	IMP M+2	Tryptophan M+2	
Carnosine M+4	IMP M+3	UDP-galactose/glucose M+1	
Citrulline M+1	IMP M+4	UDP-galactose/glucose M+2	
Citrulline M+2	Indoleacetic Acid M+1	UMP M+1	
CMP M+1	Kynurenic acid M+1	UMP M+2	
CMP M+2	Lysine M+2	Urate M+1	
CMP M+3	Methionine Sulfoxide M+1	Urate M+2	
Creatine/hydrolyzed phosphocreatine M+2	N-acetylleucine M+1	Urate M+3	
Creatine/hydrolyzed phosphocreatine M+3	N-acetylputrescine M+1	Urate M+4	
Creatinine M+1	N-acetylputrescine M+2	Xanthine M+4	
Creatinine M+2	N-acetyltryptophan M+1	XMP M+1	
Creatinine M+3	N-acetyltryptophan M+2	XMP M+2	
Cystathionine M+2	N6-acetyllysine M+1	XMP M+3	
Cysteine M+1	N6-acetyllysine M+2	XMP M+4	
