# Supplementary material for: SLC4A11 mediates ammonia import and promotes cancer stemness in hepatocellular carcinoma
Source: JCI Insight. 2024 Nov 8;9(21):e184826. doi: 10.1172/jci.insight.184826 (PMC11601557; doi:10.1172/jci.insight.184826)

Unedited blots for Supplementary Figure 5B

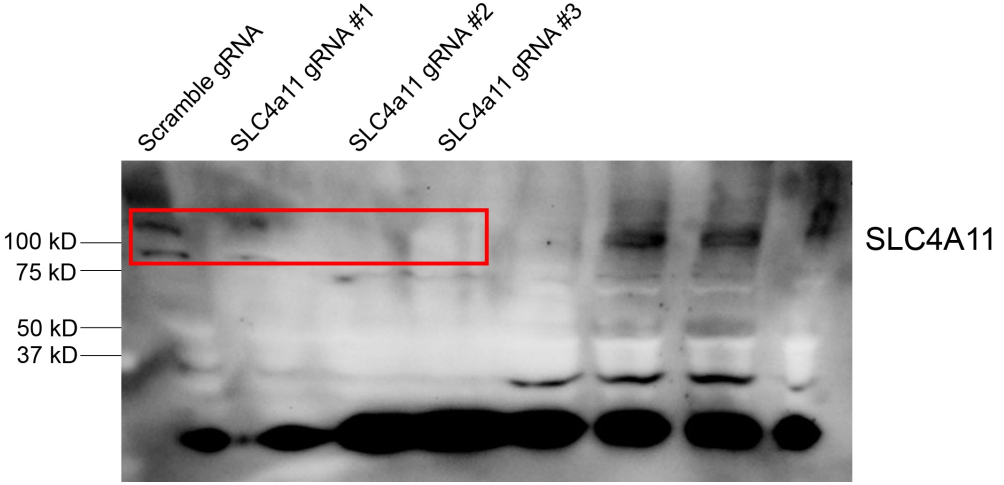

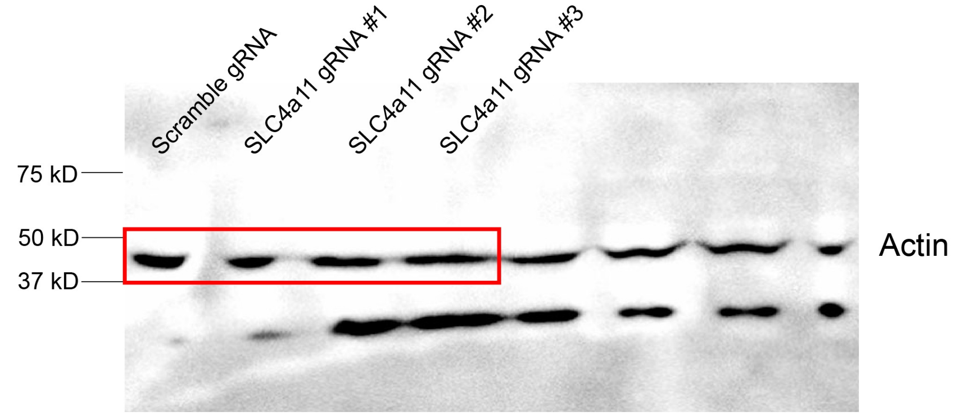

Unedited blots for Supplementary Figure 5D

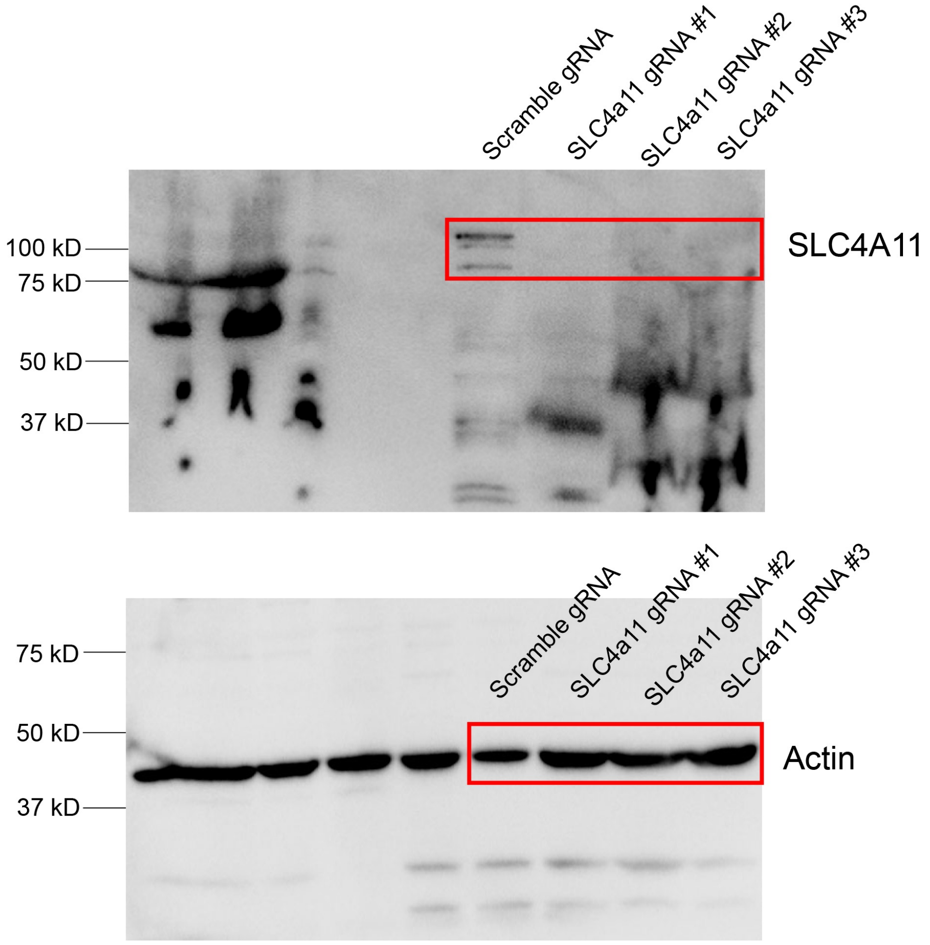

Unedited blots for Supplementary Figure 5E

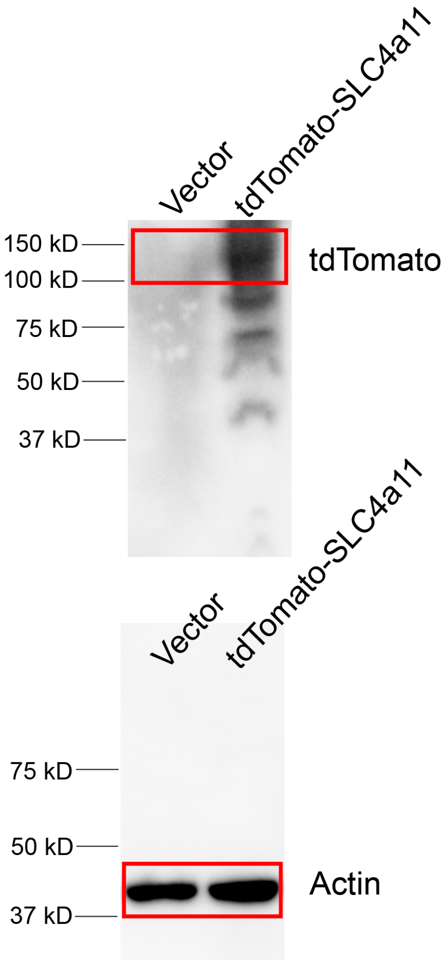

Supplement: Unedited blot and gel images [file jciinsight-9-184826-s291.pdf]
